# Uptake and Accumulation of Oxidized Low-Density Lipoprotein during *Mycobacterium tuberculosis* Infection in Guinea Pigs

**DOI:** 10.1371/journal.pone.0034148

**Published:** 2012-03-30

**Authors:** Gopinath S. Palanisamy, Natalie M. Kirk, David F. Ackart, Andrés Obregón-Henao, Crystal A. Shanley, Ian M. Orme, Randall J. Basaraba

**Affiliations:** Department of Microbiology, Immunology and Pathology, Colorado State University, Fort Collins, Colorado, United States of America; Queen Mary University of London, United Kingdom

## Abstract

The typical host response to infection of humans and some animals by *M. tuberculosis* is the accumulation of reactive oxygen species generating inflammatory cells into discrete granulomas, which frequently develop central caseous necrosis. In previous studies we showed that infection of immunologically naïve guinea pigs with *M. tuberculosis* leads to localized and systemic oxidative stress that results in a significant depletion of serum total antioxidant capacity and the accumulation of malondialdehyde, a bi-product of lipid peroxidation. Here we show that in addition, the generation of excessive reactive oxygen species *in vivo* resulted in the accumulation of oxidized low density lipoproteins (OxLDL) in pulmonary and extrapulmonary granulomas, serum and lung macrophages collected by bronchoalveolar lavage. Macrophages from immunologically naïve guinea pigs infected with *M. tuberculosis* also had increased surface expression of the type 1 scavenger receptors CD36 and LOX1, which facilitate the uptake of oxidized host macromolecules including OxLDL. Vaccination of guinea pigs with Bacillus Calmette Guerin (BCG) prior to aerosol challenge reduced the bacterial burden as well as the intracellular accumulation of OxLDL and the expression of macrophage CD36 and LOX1. *In vitro* loading of guinea pig lung macrophages with OxLDL resulted in enhanced replication of bacilli compared to macrophages loaded with non-oxidized LDL. Overall, this study provides additional evidence of oxidative stress in *M. tuberculosis* infected guinea pigs and the potential role OxLDL laden macrophages have in supporting intracellular bacilli survival and persistence.

## Introduction

It is estimated that one out of every three people worldwide is infected with *M. tuberculosis* the primary causative agent of human tuberculosis [1]. Because tuberculosis is a chronic inflammatory disease, generation of excessive reactive oxygen species (ROS) leading to a depletion of host antioxidant defenses, has been implicated in the disease pathogenesis. Markers of free radical damage such as malondialdehyde have been shown to be elevated in the peripheral circulation of human patients with active tuberculosis along with decreased serum antioxidants [2], [3]. In a previous study, we showed that *in vivo* oxidative stress in guinea pigs experimentally infected with virulent *M*. tuberculosis was also due in part to a depletion of host antioxidant defenses [4]. We also showed that treatment of *M. tuberculosis* infected guinea pigs with the antioxidant drug N-acetylcysteine reduced systemic oxidative stress by restoring the serum total antioxidant capacity, which lessened the severity of granuloma necrosis and reduced extrapulmonary dissemination of bacilli. These data suggest that inhibition of excessive ROS generation therapeutically either alone or in combination with standard drug therapy may be beneficial by limiting the progression of pulmonary and extrapulmonary tuberculosis in humans.

It has been known for nearly a century that the caseum of necrotic tuberculous granulomas are rich in host lipids [5]. Besides being an important source of energy, lipids *in* the form of cholesterol and fatty acids have been shown to facilitate mycobacterial entry and growth [6], [7], [8], [9]. Lipid-laden macrophages are a prominent and consistent feature of granulomatous lesions in animals and in infected with *M. tuberculosis* [10], [11], [12]. Recently lipid-laden macrophages have been shown to support the persistence of non-replicating bacilli and to be defective in phagocytic and bactericidal activity [13]. The diminished antimicrobial capacity of infected macrophages may enable *M. tuberculosis* to exploit this unique intracellular microenvironment for survival and replication *in vivo*.

Lipid-laden macrophages occur in a variety of infectious and non-infectious inflammatory conditions [14], [15]. Vacuolated macrophages also referred to as “foam cells” are central to the pathogenesis of atherosclerosis where oxidative stress plays a major role in disease pathogenesis. The morphologic similarities between foam cells in atherosclerosis and tuberculosis led us to hypothesize that low-density lipoproteins (LDL) are among the host macromolecules that accumulates within macrophages associated with granulomas of *M. tuberculosis* infected guinea pigs. Similar to humans, the majority of circulating cholesterol in guinea pigs is carried as LDL whereas in other rodent species, less than 50% of circulating cholesterol is in the form of LDL. Guinea pigs and humans share other similarities in cholesterol and lipoprotein metabolism, which makes them an ideal model to study lipid metabolism during *M. tuberculosis* infection. Unlike LDL which is taken up by specific macrophage LDL receptors (LDL-R), the oxidized form of LDL (OxLDL) is taken up by the scavenger receptors SR-A, SR-B1, CD36 and lectin-like oxidized low-density lipoprotein receptor-1 (LOX1) during foam cell formation in atherosclerotic lesions [16]. Scavenger receptors recognize and facilitate the uptake of macromolecules with negative charges including modified LDL. Among the multiple OxLDL receptors that have been characterized, CD36 and LOX1 are the most dominant with both being involved in the pathogenesis of cardiovascular disease and atherosclerosis [17], [18], [19], [20]. Moreover, CD36 has been shown to be involved in the uptake of *M. tuberculosis* by macrophages and non-phagocytic cells [21].

In this study, we investigated whether oxidative stress associated with experimental *M. tuberculosis* infection in guinea pigs results in the accumulation of OxLDL and increased expression of the OxLDL receptors, CD36 and LOX1. Because BCG vaccination of guinea pigs prior to challenge delays granuloma formation and reduces disease severity, we investigated whether BCG vaccination abrogates OxLDL accumulation and CD36 and LOX1 receptor expression in guinea pigs infected with the H37Rv strain of *M. tuberculosis*. Finally, we determined whether *in vitro* OxLDL loading of guinea pig alveolar macrophages altered the growth and intracellular survival of *M. tuberculosis*.

## Methods

### Ethics Statement

All experimental protocols were in accordance with the National Research Councils Guide for the Care and Use of Laboratory Animals and was approved by the Animal Care and Usage Committee of Colorado State University under protocol number 08-212A-02.

### Aerosol infection

*M. tuberculosis* H37Rv strain (TMC#102; Trudeau Institute, Saranac Lake, NY) collected and frozen at mid-log phase of growth in Proskauer-Beck liquid medium containing 0.05% Tween 80 was used for infection. A thawed aliquot of *M. tuberculosis* was diluted in sterile water to 10^6^ CFU/ml for a total working stock volume of 20 ml. Guinea pigs, approximately 9 months old, were purchased from Charles River Laboratories (North Wilmington, MA, USA) were randomly assigned to treatment groups and were exposed to aerosolized bacilli using a Madison aerosol generating chamber (University of Wisconsin Machine Shop, Madison, WI) with a starting volume of 15 ml of working stock.

### BCG vaccination

Guinea pigs were vaccinated with 1×10^4^ *Mycobacterium bovis* (BCG, strain Pasteur) or mock vaccinated with saline, intra-dermally 4 weeks prior to aerosol exposure to *M. tuberculosis*. *M. bovis* BCG was grown in Proskauer-Beck similar to *M. tuberculosis* and frozen at −80°C until used for infection [22].

### Euthanasia and sample collection

At 5, 15, 20, 30, or 90 days after infection, guinea pigs were euthanized by an overdose (1 ml per 0.75 kg body weight) of sodium pentobarbital (Sleepaway; Fort Dodge Laboratories Inc.) by intraperitoneal injection. Following euthanasia, the left pulmonary lobes were infused *in situ* with 5 ml of 4% paraformaldehyde and were allowed to fix for 48 hours and then transferred to and stored in 70% ethanol. Tissues were routinely processed by embedding in paraffin, sectioned at 5 µm, and stained with hematoxylin and eosin (H&E) [23].

### Evaluation of serum OxLDL levels

Systemic oxidized LDL levels were measured in serum collected from BCG or saline vaccinated guinea pigs at 15, 30, and 60 days after infection using a commercially available mouse anti-human copper oxidized LDL (mAb-4E6)-based competitive ELISA (Mercodia, Winston Salem NC). The specificity of mAb-4E6 (kindly provided by Mercodia) for guinea pig OxLDL was confirmed by the loss of immunoreactivity on lung tissue sections in the presence of excess antigen (human copper-oxidized LDL).

### Immunohistochemistry

Approximately 5 µm thick paraffin lung sections from infected guinea pigs were collected on positively charged glass slides, deparaffinized, rehydrated and antigen retrieval enhanced by incubating in Target Retrieval solution, pH 6.0 (DAKO, Carpentaria, CA) for 25 min at 90°C, followed by a 20 min cooling period at room temperature. Endogenous peroxidase activity was quenched with 0.3% hydrogen peroxide treatment for 15 min. The slides were subjected to two blocking steps after rinsing once in Tris buffered saline with 1% Tween-20 (TTBS): (i) 15 min incubation with 0.15 mM glycine in PBS, and (ii) 30 min incubation with 1% normal horse serum with a rinse in TTBS in between. The slides were then incubated with rabbit polyclonal antibody to human CD36, LOX1 (Santa Cruz Biotechnology, Santa Cruz, CA) and copper-oxidized LDL (Abcam, Cambridge, MA), or non-immune rabbit serum at a 1∶100 dilution in blocking buffer followed by several rinses in TTBS and incubated for 30 min. with biotinylated goat-anti rabbit-IgG (Vector Laboratories). Bound antibody was visualized using the Avidin-Biotin system (Vectastain; Vector Laboratories) and diaminobenzidine substrate (Dako; Carpentaria, CA). Sections were counterstained with Meyer's hematoxylin (Scytek Laboratories; Logan, Utah), mounted, cover slipped and examined by light microscopy. For *ex vivo* OxLDL immunocytochemistry, alveolar macrophages were collected at necropsy from *M. tuberculosis*-infected guinea pigs at by bronchioalveolar lavage as described below and cytospin preparations prepared (Thermo Scientific, Waltham, MA) on charged glass slides. Slides were air dried and fixed with 100% ice cold methanol and processed as described earlier starting with the primary antibody incubation.

The specificity of anti-human CD36 and LOX1 antibodies on guinea pig tissues was confirmed by blocking the primary antibodies with the respective purified antigens (CD36 - Affinity Bioreagents, Golden CO; LOX1 - Santa Cruz Biotechnology, Santa Cruz, CA; copper oxidized LDL - Biomedical Technologies, Stoughton, MA) at an antibody to antigen ratio of 1∶5. Photomicrographs were acquired with an Olympus DP70 camera and associated computer software. Microscopic lesions stained with H&E and sections stained by immunohistochemistry, were scored by the reviewer blinded to the treatment groups.

### Lesion analysis

To evaluate the progression of disease over time, a previously described histological grading system was used [24]. Lung sections were scored based on the following six criteria: (i) percent of lung affected were ranked at low magnification based on the percent of the lung affected by lesion as follows: 0-no lesions, 1, ≤25% of lung involved, 2, 25–50% of lung involved, 3, 50–75% of lung involved, 4, >75% of lung involved. (ii) primary lesions: 0-no primary lesions present, 1, a single primary lesion, 2, two or more multi-focal primary lesions, 3, two or more multifocal to coalescing primary lesions, 4, multiple coalescing and extensive primary lesions. (iii) secondary lesions: 0, no secondary lesions present, 1, <25% of lung involved, 2, 25–50% of lung involved, 3, 50–75% of lung involved, 4, >75% of lung involved. Necrosis (iv), mineralization (v) and fibrosis (vi) were scored based on severity as follows: 0, none, 1, minimal, 2, mild, 3, moderate, 4, marked. Subcategory scores were added for the final total score for each organ (Range: 0–24).

### Immunohistochemical scoring

For immunohistochemical scoring, the total lung area was divided into primary lesions (lesions with a central core of necrosis) and primary lesion free (PLF) lungs. Within these regions the overall immunohistochemical scoring was determined based on extent of staining (0, none; 1, <25%; 2, 25–50%; 3, 50–75%; 4, >75%) and staining intensity (0, none: 1, mild; 2, moderate; 3, marked; 4, extensive). The resultant total overall scores were converted to a four-point scale.

### Isolation of guinea pig alveolar macrophages

Guinea pig alveolar macrophages were collected from naive, non-infected guinea pigs by repeated infusion and aspiration of 10 ml of 1× Hank's balanced salt solution (MP Biomedicals, Solon, OH) three times. Cells were pelleted by centrifugation and washed twice with 1× PBS and the cell numbers and viability determined using a hemocytometer in combination with Trypan blue dye exclusion (Gibco Life Technologies, Grand Island, NY). The numbers of viable macrophages were adjusted to 2×10^5^ cells/ml in a serum free media (Aim-V; Gibco, Grand Island, NY) containing bovine serum albumin, L-glutamine, streptomycin sulfate, and gentamicin sulfate.

### *In vitro* culture of guinea pig alveolar macrophages

Bronchoalveolar lavage cells (2×10^5^/well) were allowed to adhere for 1 hour to each well of a 24 well flat-bottom plate (Becton Dickinson Labware, Franklin Lakes, NJ). Media and non-adherent cells were removed and the monolayers washed with 1× PBS. Representative wells were stained with Diff-Quik staining (Dade Behring, Newark, DE) and the staining revealed over 95% cells to have the morphologic characteristics of alveolar macrophages. Adherent alveolar macrophages that were treated with either non-oxidized low density lipoprotein or copper-oxidized low density lipoprotein (Biomedical Technologies, Stoughton, MA) at a dose of 25 µg/ml then infected or mock-infected with *M. tuberculosis* H37Rv (MOI 5∶1). At 4 hrs, 24 hrs, 3 days and 7 days after the infection, one set of wells were analyzed for cell viability using CellTitre 96 Acqueous solution cell proliferation assay (Promega, Madison, WI) following manufacturers protocol and from another set of wells, the adherent macrophages were washed and gently scraped from the surface and resuspended 1× PBS. The macrophages were then homogenized, plated on nutrient 7H11 agar plates following serial dilution and *M. tuberculosis* numbers determine after three weeks of culture at 37°C and data expressed as colony-forming units.

Different set of the primary alveolar macrophages from the same experiment but grown in eight well chamber glass slides were fixed with 10% methanol. Cells were permeabilized with 0.1% Triton X-100 and endogenous peroxidase activity was blocked with 0.3% hydrogen peroxide. Cells were stained with diluted Rhodamine B (Sigma, St Louis, MO) for 30 minutes (for *M. tuberculosis*) and de-stained twice with an acid alcohol solution. Slides were then blocked using Tyramide Signal Amplification (TSA kit; Invitrogen, Carlsbad, CA) blocking Solution. Primary polyclonal rabbit anti-OxLDL (Calbiochem, San Diego, CA) antibody was added to the slides and the slides were incubated overnight at 4°C. After washing, slides were incubated at room temperature with the TSA goat anti-rabbit IgG HRP for 1 hour. TSA Alexa Fluor 488 tyramide and 0.0015% hydrogen peroxide is added for detection of antibody complex. Slides were counterstained with diluted Hoechst in 1× PBS. Slides were mounted using ProLong Gold (Invitrogen, CA). Images were taken on a Nikon Eclipe 80i with DAPI, FITC and TRITC cubes and composite images produced with Adobe Photoshop 7.0.

### Statistical analysis

The statistical differences of lesion or immunostaining scores among different time points were analyzed using non-parametric Kruskal-Wallis one-way analysis of variance. The specific statistical differences between two individual group scores were analyzed using the Mann-Whitney t test. The statistical differences between serum OxLDL levels were analyzed using two-way ANOVA and non-paired student t test. Statistical analysis of correlation was performed using Pearson's correlation method and coefficient of correlation for the pairs of groups discussed in the results. All of the statistical tests were done using the Prism software (version 4.03, Graph Pad, La Jolla, CA).

## Results

### Lung lesion burden during *M. tuberculosis* infection

**Figure 1:**
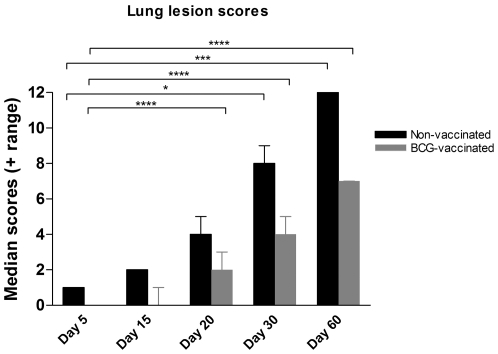
*M. tuberculosis* infection of guinea pigs results in progressive lung lesions that are less severe in BCG vaccinated animals. The lung lesion burden increases with time in guinea pigs infected by aerosol with the H37Rv strain of *M. tuberculosis*. BCG vaccination prior to challenge decreases the rate and severity of lung granulomas as determined by lesion scores. The bars represent median values plus range (when present) for each group (n = 5). The asterisks denote statistically significant increase compared to day 5 after infection (* = p<0.05, *** = p<0.001 and **** = p<0.0001).

The histopathologic changes within the lungs of *M. tuberculosis*-infected guinea pigs were quantified using a scoring system that correlates disease progression with lesion burden or immunohistochemical scores. Figure 1 shows data the median lung lesion scores (n = 5) on days 5, 15, 20, 30 and 60 after infection in both saline-vaccinated and BCG-vaccinated guinea pigs. The number and extent of the lung lesions progressed rapidly from 5 to 60 days and lesion burden at days 30 and 60 of infection were statistically significant from day 5 values. Lesions were less severe and disease progression was delayed in guinea pigs vaccinated with BCG prior to virulent challenge.

### Serum OxLDL levels

**Figure 2:**
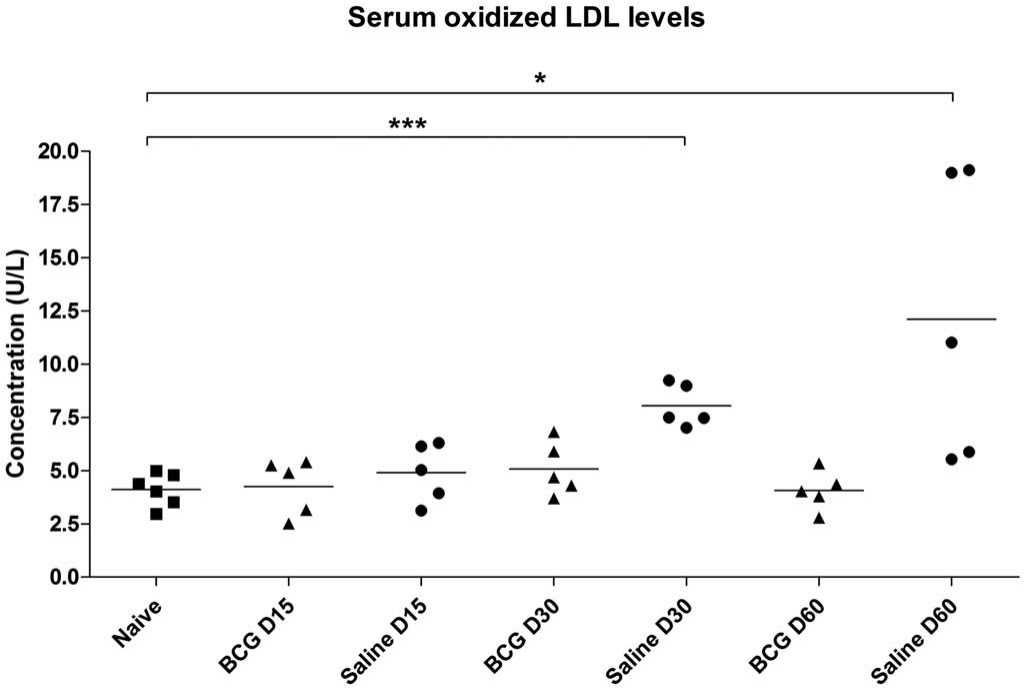
Serum OxLDL levels increase in guinea pigs infected with *M. tuberculosis* infection. In guinea pigs sham-vaccinated with saline, serum OxLDL levels increased with the progression of disease as determined by a competitive ELISA. BCG vaccination of guinea pigs prior to virulent challenge abrogated the increase in serum OxLDL levels. Data is expressed as the mean values for each treatment group (n = 5). The asterisks denote statistically significant increase compared to the naive animals (* = p<0.05 and *** = p<0.001).

Serum OxLDL levels were measured by ELISA in uninfected guinea pigs and in BCG or saline vaccinated animals on days 15, 30 and 60 of infection. Serum OxLDL levels increased significantly in the mock-vaccinated animals on days 30 and 60 (p<0.05) compared to the BCG-vaccinated animals (Figure 2). The increase in the serum OxLDL levels correlated with the progression of disease expressed by lesion scores (r = 0.96). The highest serum OxLDL levels were observed in three of five animals on day 60 of the infection. Despite the elevation in serum OxLDL levels on day 60, there was no significant correlation between bacterial burden in lungs, spleen and lymph node or the extent of histopathologic lesion severity. However, the progressive increase in mean OxLDL levels and median lung lesion scores correlated on days 30 and 60. BCG vaccination abrogated the OxLDL increase in *M. tuberculosis* infected animals even out to 60 days of infection.

### OxLDL expression in lungs

**Figure 3:**
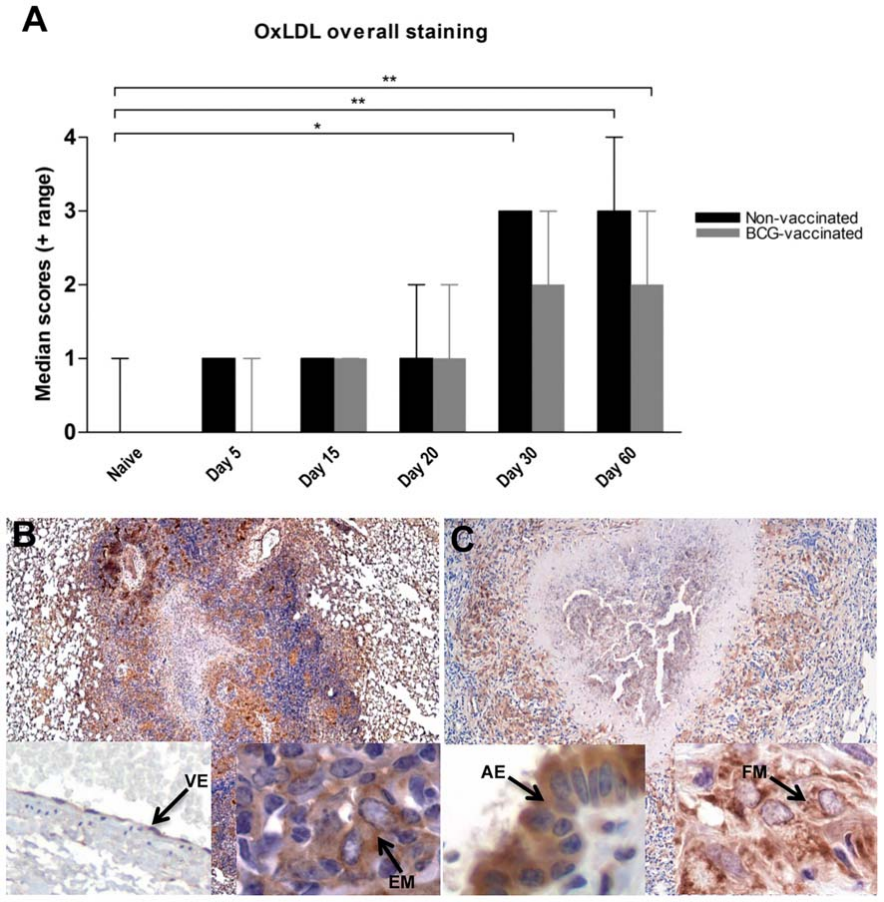
OxLDL is concentrated and accumulates in macrophages within lung granulomas of *M. tuberculosis* infected guinea pigs. A. The expression of OxLDL in the lungs lesions increased with the progression of disease in guinea pigs sham vaccinated with saline and BCG vaccinated animals as determine by scoring of immunostained sections. The bars represent median values plus range (when present) for each group (n = 5). The asterisks denote statistically significant increase compared to the naive animals (* = p<0.05 and ** = p<0.01). The photomicrographs B and C represent immunostaining of OxLDL predominantly within primary granulomas at 30 and 60 days of infection respectively (40×). The inserts (1000×) show intracellular staining within vascular endothelial cells (VE), epithelioid macrophages (EM), airway epithelial cells (AE) and foamy macrophages (FM).

Oxidized LDL, formed during oxidative stress conditions, is taken up and accumulates within the cytoplasm of macrophages and other cell types at the site of chronic inflammation [25]. To determine whether lung accumulation of OxLDL was diffuse or restricted to a particular lesion type, tissue sections were scored as total or overall staining or were scored based on the extent of involvement of primary or secondary granulomas and uninvolved lung, designated primary lesion free lung (PLF). Overall immunostaining for OxLDL in lung sections from *M. tuberculosis*-infected guinea pigs increased gradually (Figure 3A) as the infection progressed and similar to the serum levels, correlated with disease progression (r = 0.99). The lungs from non-infected guinea pigs had minimal OxLDL accumulation in the normal lung parenchyma (Figure 3E). Statistically significant increases in OxLDL staining were seen on days 30 and 60 of the infection (p<0.01 for both) with the majority of the OxLDL staining being restricted to the primary granulomas (Figure 3B). There were no significant changes in the expression of OxLDL within the PLF lung. OxLDL expression was most prominent in lesion-associated macrophages including those with vacuolated cytoplasm (foam cells), airway epithelial cells, vascular endothelial cells and granulocytes within the inflammatory zone of the primary granulomas with minimal staining within the necrotic center (Figure 3E and F).

**Figure 4:**
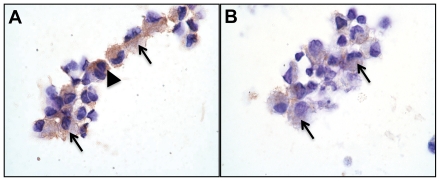
OxLDL accumulates in pulmonary alveolar macrophages of *M. tuberculosis*-infected guinea pigs. A and B represent OxLDL immunostaining in BAL cells collected from *M. tuberculosis* infected guinea pigs at day 30 and day 60 after infection respectively (1000× magnification). Predominantly macrophages (arrows) and occasionally granulocytes (arrowhead) show intracellular staining.

Guinea pig lung cells collected by bronchoalveolar lavage also showed an increase in accumulation of cytoplasmic OxLDL by days 30 and 60 of infection (Figures 4A and B respectively). The BAL cells were predominantly alveolar macrophages intermixed with fewer numbers of granulocytes in which OxLDL expression levels were similar to that seen for macrophages. BAL cells from non-infected animals showed minimal to no OxLDL staining (data not shown).

### CD36 expression in lungs

**Figure 5:**
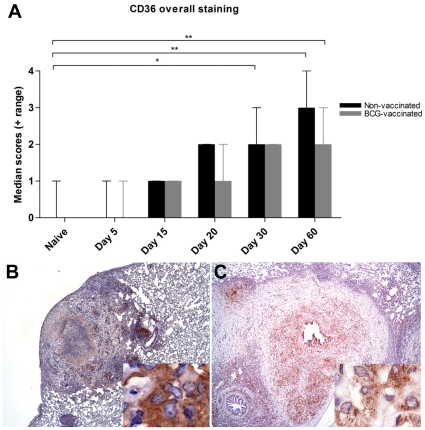
Lung lesions in *M. tuberculosis* infected guinea pigs have increased expression of CD36. A. The overall expression of CD36 in the lungs increases with time in guinea pigs sham-vaccinated with saline or BCG as determined by scoring of immunostained sections. The bars represent median values plus range (when present) for each group (n = 5). The asterisks denote statistically significant increase compared to the naive animals (* = p<0.05 and ** = p<0.01). The photomicrographs B and C represent immunostaining of CD36 predominantly within lung granulomas at 30 and 60 days of infection respectively (40×). The inserts (1000×) show intracellular staining within macrophages.

The scavenger receptor CD36 is a receptor for OxLDL and is expressed on a wide range of cell types including macrophages. With the progressive increase in OxLDL expression in the lungs of *M. tuberculosis* infected guinea pigs, we used immunohistochemistry to determine whether there was a corresponding increase in the OxLDL receptor CD36. Overall CD36 expression in the lungs from *M. tuberculosis*- infected guinea pigs increased gradually as the infection progressed (Figure 5A). The increase in receptor expression levels correlated (r = 0.99) with the increase in the lung lesion scores. Increases in CD36 expression were statistically significant on days 30 and 60 of infection (p<0.05 and 0.01 respectively). Non-infected guinea pigs expressed minimal to no CD36 staining. The increase in the CD36 expression was predominantly within the inflammatory zone of the primary granulomas (Figure 5B) similar to the pattern seen for OxLDL. In contrast, the expression within the secondary lesions and non-affected lung was unchanged even out to 60 days of infection (Figure 5.5C). Despite the reduced lesion burden seen in BCG vaccinated guinea pigs, there was an increase in CD36 expression by day 60 of infection. The cell types that showed the most intense CD36 expression were macrophages including those with vacuolated cytoplasm, airway epithelial cells, endothelial cells and granulocytes (Figure 5E and F).

### LOX1 expression in lungs

**Figure 6:**
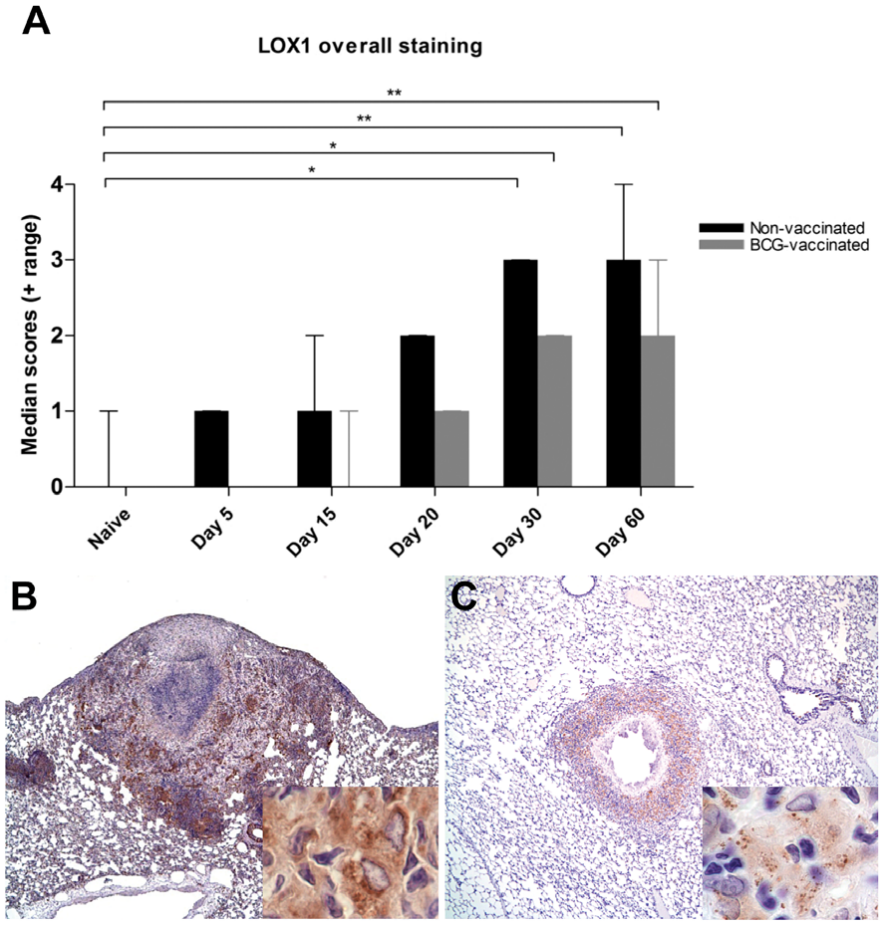
Lung lesions in *M. tuberculosis* infected guinea pigs have increased expression of LOX1. A. The overall expression of LOX1 in the lungs increases with time in guinea pigs sham-vaccinated with saline or BCG as determined by scoring of immunostained sections. The bars represent median values plus range (when present) for each group (n = 5). The asterisks denote statistically significant increase compared to the naive animals (* = p<0.05 and ** = p<0.01). The photomicrographs B and C represent immunostaining of LOX1 predominantly within lung granulomas at 30 and 60 days of infection respectively (40×). The inserts (1000×) show intracellular staining within macrophages.

LOX1 is another major receptor for oxidized LDL and is known to be transcriptionally up-regulated during various inflammatory diseases that have oxidative stress as a central pathogenesis [26]. Similar to CD36, we sought to evaluate the LOX1 receptor expression levels in the lungs *M. tuberculosis* infection in guinea pigs using immunohistochemistry. Similar to OxLDL and C36, overall LOX1 protein expression levels increased significantly as the infection progressed (Figure 6A), a rate that correlated (r = 0.99) with the increase in the lung lesion scores. Lungs from non-infected guinea pigs had minimal to no LOX1 expression by immunohistochemistry. This increase in LOX1 expression was statistically significant on days 30 and 60 after infection (p<0.05 and 0.01 respectively). Similar to CD36 expression, the increase in the LOX1 immunostaining occurred predominantly within the inflammatory zone of primary granulomas (Figure 6B) with minimal staining of the cellular debris within the necrotic center. The staining was comparatively unchanged within the PLF lung areas at different time points (Figure 6C). The pattern of staining for LOX1 was significantly different than for CD36. Whereas CD36 showed a diffuse staining pattern, macrophages expressing LOX1 had discrete cytoplasmic stippling that was most prominent by day 60 (Figure 6E and F). Similar to OxLDL and CD36, BCG vaccination prior to virulent challenge reduced the extent of LOX1 expression but increases at days 30 and 60 of infection were significantly higher than days 5, 15 and 20.

### Enhanced bacterial survival in OxLDL-loaded macrophages

**Figure 7:**
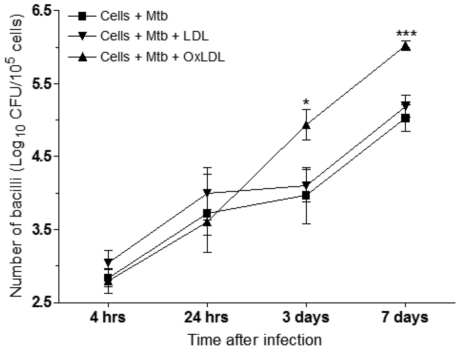
OxLDL loading of guinea pig pulmonary alveolar macrophages results in increased mycobacterial survival. Normal guinea pig alveolar macrophages were obtained by bronchoalveolar lavage and loaded with oxidized or non-oxidized LDL prior to in vitro infection with the H37Rv strain of M. tuberculosis. The data represents the mean CFU (± SD) recovered from infected cultures from 4 hours to 7 days of infection from 4 separate experiments (n = 4). The asterisks denote statistically significant increase compared to the control group at respective time points (* = p<0.05 and *** = p<0.001).

To determine whether OxLDL accumulation in alveolar macrophages influenced the growth of *M. tuberculosis*, we treated primary guinea pig alveolar macrophages with purified OxLDL prior to *in vitro* infection. Macrophages were either treated with non-oxidized low-density lipoprotein or copper-oxidized low-density lipoprotein prior to *in vitro* infection. One group of macrophages did not receive any treatment prior to infection. Our results showed an approximately 10 fold (p<0.005) increase in bacilli in OxLDL-treated macrophages at days 3 and 7 after the initial infection (Figure 7) compared to non-treated macrophages or those pre-treated with non-oxidized lipoproteins. All bacterial counts were normalized to 10^5^ cells for all time points and groups. No statistically significant differences in the viability of macrophages were noted between different treatment groups at all time points examined (data not shown) suggesting that the observed differences in bacterial numbers were not related to the differences in the viability of macrophages.

**Figure 8:**
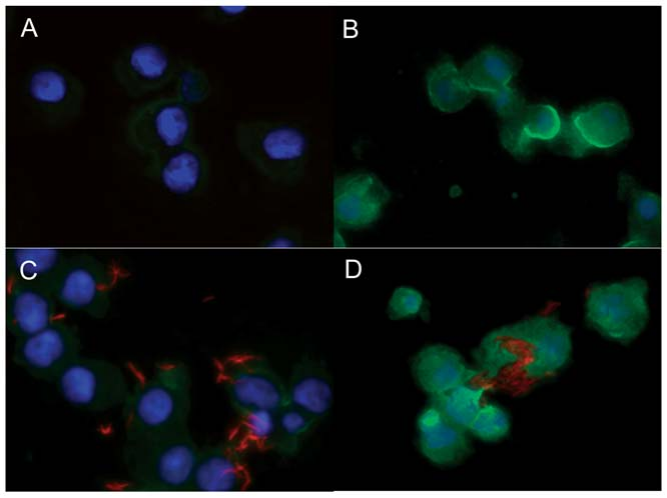
OxLDL loaded guinea pig pulmonary alveolar macrophages support intracellular growth of *M. tuberculosis in vitro*. A. Immunofluorescence of normal guinea pig alveolar macrophages show no evidence of OxLDL cytoplasmic staining. B. Uninfected alveolar macrophages loaded with OxLDL show strong cytoplasmic immunofluorescence staining after 3 days of treatment. C. Alveolar macrophages loaded with LDL for 3 days and infected with *M. tuberculosis* (stained with rhodamine/auramine) show minimal to no positive staining for OxLDL. D. Co-localization of *M. tuberculosis* stained with rhodamine/auramine within alveolar macrophages loaded with OxLDL for 3 days *in vitro*.

To confirm the uptake and accumulation of OxLDL by the primary alveolar macrophages, cells were stained with OxLDL by immunofluorescence. Our results showed significant cytoplasmic accumulation of OxLDL in the treatment groups (Figure 8B, 8D). In *M. tuberculosis* infected group, bacteria were present within the cytoplasm of cells co-expressing OxLDL (Figure 8D). No noticeable expression of OxLDL was present in the untreated groups including the group that received LDL despite the presence of intracellular bacilli (Figures 8A, 8C).

## Discussion

Our data builds on our previous study that showed that systemic and pulmonary oxidative stress is a prominent feature of experimental tuberculosis in guinea pigs [4]. The major findings in this study was that not only does OxLDL accumulate in the serum and within granulomas of *M. tuberculosis*-infected guinea pigs but the expression of the scavenger receptors CD36 and LOX1 were also increased with similar kinetics and relative magnitude as OxLDL, especially within primary lesion with necrosis. Even though CD36 and LOX1 bind multiple ligands, the elevation of OxLDL levels during *M. tuberculosis* infection suggests that these receptors may play an important role in oxidized lipid uptake and accumulation. Unlike the other macrophage scavenger receptor types A-I and II (SR-AI/II) that recognize copper-oxidized and acetylated-LDL, CD36 can also recognize LDL modified by myeloperoxidase-hydrogen peroxide-nitrite system (MPO-OxLDL) which also has physiological relevance associated with chronic inflammation [27]. Recently we have showed that the ability of *M. tuberculosis* to resist copper accumulation is critical for virulence and that necrotic granulomas in guinea pigs accumulate significant amounts of the free radical generating transition metals copper and iron *in vivo* [28], [29]. These data support the biological relevance and potential role free radical generating transition metal may have in modifying host and possibly bacilli macromolecules.

In an earlier study, we showed that malondialdehyde (MDA), a byproduct of lipid peroxidation, accumulated in a variety of cell types early in *M. tuberculosis* infected guinea pigs [4]. Further evidence of excessive ROS generation during infection was a marked depletion of serum total antioxidant capacity and specifically the critical antioxidant tripeptide glutathione [4]. Tuberculosis is a chronic inflammatory disease characterized by systemic oxidative stress with elevated markers of free radical damage in patients with active disease [2], [3]. Considering a variety of host macromolecules such as proteins, lipids and nucleic acids that are susceptible to ROS mediated damage, it is not surprising that oxidatively modified LDL is among the host macromolecules that accumulate during experimental *M. tuberculosis* infection in guinea pigs. In addition, OxLDL contains other lipid peroxidation products that may also contribute directly to the pathogenesis of granuloma necrosis [30], [31].

Unlike LDL which is quickly degraded, and does not accumulate within cells, OxLDL is stored and accumulates within cells especially macrophages [32]. The lipid components of OxLDL includes esterified and non-esterified cholesterol, phospholipids and triglycerides that make up approximately 80% of the total LDL content [33], [34]. As cholesterol and other host lipids may serve as a carbon source for *M. tuberculosis* [35], we determined whether these oxidized lipids were among the stored products associated with vacuolated macrophages *in vitro* and *in vivo*. A unique subpopulation of macrophages with abundant, vacuolated cytoplasm is a prominent feature of chronic inflammatory lesions like tuberculosis and atherosclerosis. These data indicate however that OxLDL expression was not restricted to foamy macrophages since epithelioid cells had similar staining characteristics as demonstrated by immunohistochemistry. This discrepancy could reflect the degree and duration of the lipid accumulation over the course of the disease with foamy macrophages representing mature or more chronic accumulations [36]. OxLDL staining was also observed in airway epithelial and vascular endothelial cells especially in the early time points. This observation may have important implications in ROS-mediated damage to other cell types during the early phase of disease. Endothelial cells are particularly susceptible to oxidative injury. Ongoing studies in our laboratory indicate that in the early stages of granuloma formation, there is loss of vascular integrity within the inflammatory zone of primary granulomas, which may contribute to the pathogenesis of caseous necrosis.

Besides the accumulation of OxLDL, we also show that two OxLDL receptors, CD36 and LOX1 show increased expression over the course of *M. tuberculosis* infection. The scavenger receptor CD36 may have several important functions in the pathogenesis of tuberculosis. Previous studies have shown that CD36 functions in the uptake of *M. tuberculosis* by macrophages [21]. Increased CD36 receptor expression by platelets has been shown to be pro-thrombotic in several mouse models of microvascular thrombosis [37]. Because CD36 is an important receptor for the uptake of OxLDL in other diseases, the increased expression of CD36 and OxLDL in *M. tuberculosis* infected guinea pigs may reflect their importance in the pathogenesis of tuberculosis as well. Data from these studies suggest that the parallel expression of CD36 and LOX1 may function to bind and facilitate the uptake of OxLDL generated during the oxidative stress conditions associated with *M. tuberculosis* infections as is described for other chronic inflammatory diseases [18], [25]. In addition, both CD36 and LOX1 have been shown to serve as the receptor for advanced glycation end products (AGEs) which form following the non-enzymatic glycation of proteins, lipids and nucleic acids [38], [39]. The formation of AGEs is promoted during oxidative stress conditions and have been shown to accumulate in human tuberculosis lesions [40]. Recent studies in our laboratory have shown that AGEs accumulate within necrotic lesions and within the peripheral circulation of guinea pigs infected with *M. tuberculosis* (unpublished data). Collectively, these data suggest a number of pathways in which OxLDL and scavenger receptor expression on macrophages and other cells might be involved in tuberculosis granuloma pathogenesis [41], [42]. Besides OxLDL, oxidation of cellular debris containing various fatty acids may also contribute to foam cell formation. However, the contribution from oxidized fatty acids to foam cell formation is unclear. While the scavenger receptor pathway has been shown to result in uptake and stable accumulation of lipid components of OxLDL, the uptake pathway and subsequent stable accumulation of oxidized fatty acids has not been well established.

Vaccination of guinea pigs with BCG prior to infection significantly delayed the increase in serum and lesion OxLDL levels compared to animals that were mock-vaccinated with saline. BCG vaccinated guinea pigs maintained serum OxLDL levels within the range of normal, uninfected controls. However, BCG was less effective at preventing the accumulation of OxLDL as well as CD36 and LOX1 receptor expression within lung granulomas. In all cases, BCG vaccinated animals had a progressive accumulation of OxLDL, CD36 and LOX1 by day 60 that was significantly increased above values at day 5 of infection. These data are consistent with the ability of BCG vaccination to delay but not prevent the progression of pulmonary and extra-pulmonary disease [43], [44].

Our *in-vitro* study using alveolar macrophages confirms the ability of guinea pig alveolar macrophages to bind and accumulate OxLDL. The reason OxLDL treated macrophages favored bacilli growth is unknown but may be related to the utilization of intracellular host lipids especially cholesterol as an energy source [7]. Increased, albeit seemingly counter-intuitive, replication of mycobacteria noted in guinea pig broncho-alveolar macrophages laden with OxLDL conflicts with the proposed dynamic hypothesis of latent tuberculosis. Our *in-vitro* system likely represents early events in the evolution of foamy macrophages in which increased bacterial replication and resultant macrophage necrosis favor the spread of bacteria and the establishment of active disease. During the chronic stages of disease amidst high oxidative stress state in guinea pigs, mycobacteria exposed to low oxygen concentrations may become less metabolically active and persist in a non-replicating state within the foamy macrophages.

It is not clear whether activated foamy macrophages take up non-replicating bacteria within the granuloma or bacteria become non-replicating once taken up by cells in chronic stages of infection. Further studies are needed to address this question. One reason for increased bacterial survival in our *in-vitro* model is potential decreased bactericidal activity of the foamy macrophages. Evolution of foamy macrophages is likely a long-term process in which increasing amounts of host lipids accumulate within persistent lesions. Our data supports this sequential evolution theory since OxLDL also accumulates in non-vacuolated epithelioid macrophages early in the disease.

It is unclear why oxidized LDL was more effective at supporting intracellular bacilli survival and replication compared to the non-oxidized lipoprotein. The enhanced growth of *M. tuberculosis* in OxLDL loaded macrophages illustrates yet another specific role oxidative stress plays in the pathogenesis of tuberculosis. We observed only minimal foam cell formation of broncho-alveolar macrophages infected in vitro with *M. tuberculosis* (data not shown). The potential role for apoptotic cellular debris contributing to foam cell formation as demonstrated by D'Avila et al [45] was not considered in our *in vitro* model since cultures are enriched for macrophages (over 99%) with only rare granulocytes.

Overall, the findings of these studies demonstrate uptake and accumulation of OxLDL by macrophages expressing the scavenger receptors CD36 and LOX1 during *M. tuberculosis* infection in guinea pigs. Elevated receptor expression and uptake and storage of OxLDL may contribute to tuberculosis pathogenesis by altering phagocytic/bactericidal activity of lipid-laden macrophages and thus supporting increased bacterial growth in the early stages of infection [13]. The pathway of CD36 or LOX1-mediated accumulation of OxLDL could provide attractive targets for the development of novel therapeutics that interfere with this pathway to host tissue damage and conditions that favor *M. tuberculosis* replication or persistence.
